# Multi-Omics Data Fusion for Cancer Molecular Subtyping Using Sparse Canonical Correlation Analysis

**DOI:** 10.3389/fgene.2021.607817

**Published:** 2021-07-22

**Authors:** Lin Qi, Wei Wang, Tan Wu, Lina Zhu, Lingli He, Xin Wang

**Affiliations:** ^1^Department of Biomedical Sciences, City University of Hong Kong, Shenzhen, China; ^2^Key Laboratory of Biochip Technology, Biotech and Health Centre, Shenzhen Research Institute, City University of Hong Kong, Shenzhen, China

**Keywords:** multi-omics, data fusion, cancer subtyping, canonical correlation analysis, ovarian cancer, breast cancer

## Abstract

It is now clear that major malignancies are heterogeneous diseases associated with diverse molecular properties and clinical outcomes, posing a great challenge for more individualized therapy. In the last decade, cancer molecular subtyping studies were mostly based on transcriptomic profiles, ignoring heterogeneity at other (epi-)genetic levels of gene regulation. Integrating multiple types of (epi)genomic data generates a more comprehensive landscape of biological processes, providing an opportunity to better dissect cancer heterogeneity. Here, we propose sparse canonical correlation analysis for cancer classification (SCCA-CC), which projects each type of single-omics data onto a unified space for data fusion, followed by clustering and classification analysis. Without loss of generality, as case studies, we integrated two types of omics data, mRNA and miRNA profiles, for molecular classification of ovarian cancer (*n* = 462), and breast cancer (*n* = 451). The two types of omics data were projected onto a unified space using SCCA, followed by data fusion to identify cancer subtypes. The subtypes we identified recapitulated subtypes previously recognized by other groups (all *P*- values < 0.001), but display more significant clinical associations. Especially in ovarian cancer, the four subtypes we identified were significantly associated with overall survival, while the taxonomy previously established by TCGA did not (*P-* values: 0.039 vs. 0.12). The multi-omics classifiers we established can not only classify individual types of data but also demonstrated higher accuracies on the fused data. Compared with iCluster, SCCA-CC demonstrated its superiority by identifying subtypes of higher coherence, clinical relevance, and time efficiency. In conclusion, we developed an integrated bioinformatic framework SCCA-CC for cancer molecular subtyping. Using two case studies in breast and ovarian cancer, we demonstrated its effectiveness in identifying biologically meaningful and clinically relevant subtypes. SCCA-CC presented a unique advantage in its ability to classify both single-omics data and multi-omics data, which significantly extends the applicability to various data types, and making more efficient use of published omics resources.

## Introduction

It has been recognized that cancers are heterogeneous diseases comprising multiple subtypes with distinct molecular properties associated with discrepant clinical outcomes. In the last decade, tremendous efforts have been made in identifying cancer molecular subgroups (Zhao et al., 2019). Unlike traditional cancer classification based on histopathological characteristics or individual mutations, these studies employed unsupervised classification to identify biologically coherent subgroups. However, the pre-existing studies were mostly based on single-omics data, especially transcriptomic data, ignoring molecular heterogeneity occurring at other (epi-)genetic levels of gene regulation such as copy number variation, and DNA methylation. Recent advances in high-throughput biotechnologies, especially next-generation sequencing technologies, made it possible to generate (epi)genomic profiles at a significantly reduced cost, providing an opportunity for integrative analysis of multiple types of omics data. Genome-wide, multi-omics profiles of tissue samples from large-scale patient cohorts enabled a more comprehensive dissection of cancer molecular heterogeneity. International consortia such as the cancer genome atlas (TCGA) have assembled multiple cancer data types from 1,000 patients, making integrative methods essential for a better understanding of cancer biology. However, due to the difference in data scale, the complexity of dimensionality, effective integration of multi-omics data for cancer subtyping remains a significant challenge (Bersanelli et al., 2016).

To address the challenge, several computational models have been proposed, which showed promising performance. For instance, non-negative matrix factor (NMF) can be used to project multi-omics data onto dimension-reduced space for integration based on non-negative matrix decomposition (Zhang et al., 2011, 2012). However, the prerequisite of non-negative matrices needs to be satisfied, and proper normalization of the input data is crucial. Joint and individual variation explained (or JIVE) can also be used for integrative analysis of multi-omics data by quantifying the joint variation between data types followed by decomposition to reduce the dimensionality. The application of JIVE to glioblastoma showed better characterizations of different subtypes, but the robustness remains a concern due to potential outliers affecting the factorization based on principal component analysis (PCA) (Lock et al., 2013). iCluster (Shen et al., 2009) and its extensions iClusterPlus (Kirk et al., 2012) learned a joint latent variable model for integrative clustering on multiple types of data. Despite the widely demonstrated usefulness, the scalability of iCluster and its related methods to a genome-wide scale was questionable (Shen et al., 2009). Wang et al. (2014) developed a novel bioinformatic approach named “similarity network fusion (SNF)”, which iteratively fused similarity networks constructed from each type of single-omics data into a similarity network by a nonlinear combination method. SNF showed better performance than single-omics methods in cancer subtyping, as demonstrated in multiple case studies (Wang et al., 2014). However, iCluster and SNF do not provide a classification framework, and they both rely on a complete dataset of multi-omics profiles for the clustering of new samples, which is often not available, and significantly limiting their general applicability (Wang et al., 2014).

To overcome the above-mentioned challenges, we propose to fuse different types of omics data for clustering and classification of tumor samples by canonical correlation analysis (CCA) (Hotelling, 1936), a classical statistical analysis method used in multi-views biometric identification. The CCA algorithm measured the correlation between two sets of multi-dimensional data and projected onto a unified space in which the transformed vectors are maximally correlated. However, the classical CCA could not be easily applied to analyze high-throughput data in which the number of variables is much larger than the number of samples. PCA was commonly used to reduce dimensions but may discard important information of correlation and discrimination for 1,000 of variables (Witten et al., 2009). Sparse CCA solved the problem by employing singular value decomposition, seeking sparsity in both sets of variables simultaneously (Witten et al., 2009). The efficiency of SCCA (Sparse CCA) had been demonstrated in simulated genomic data in previous studies (Witten et al., 2009), providing a rationale for us to employ SCCA for cancer subtyping analysis.

**FIGURE 1:**
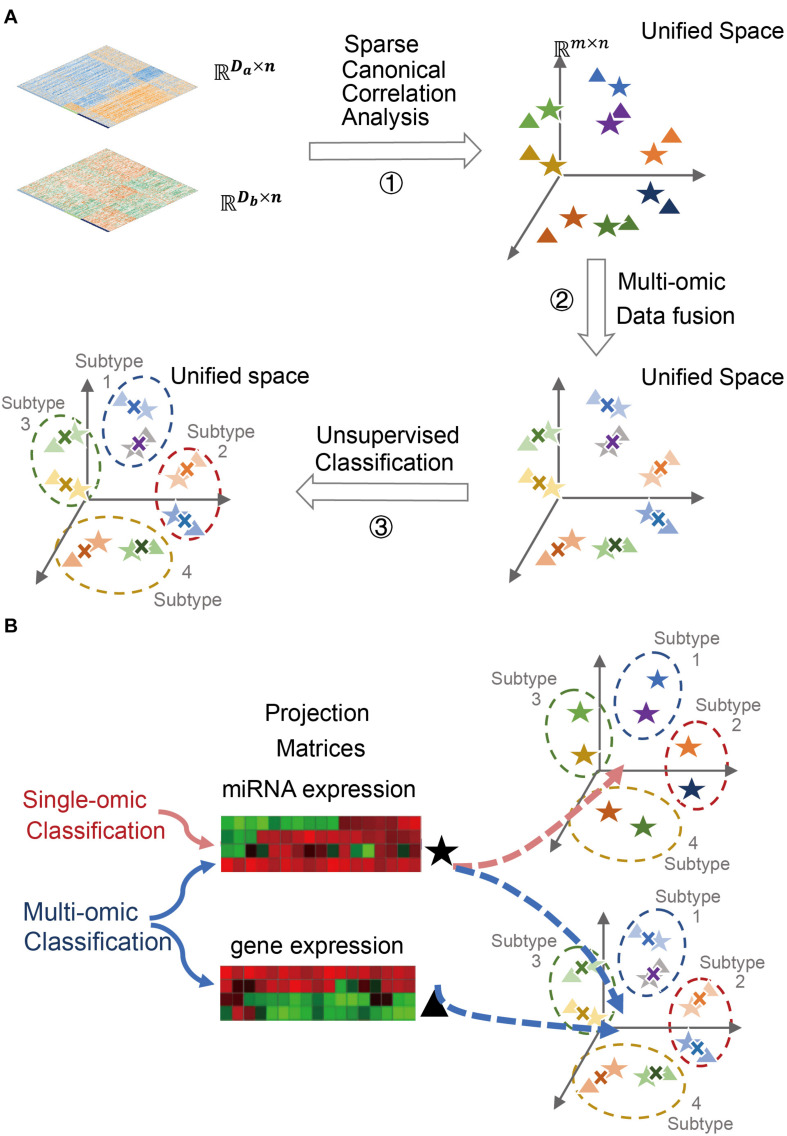
Cancer subtyping and classification using SCCA-CC. **(A)** A schematic figure illustrating the three major steps for multi-omics cancer subtyping. A toy example is used to illustrate the projection of mRNA and miRNA expression data of the same set of patient samples onto lower-dimensional unified space by sparse canonical correlation analysis (SCCA), followed by data fusion and unsupervised classification. **(B)** A schematic figure illustrating the versatile classifier can not only classify fused multi-omics data, but also individual single-omics data.

In this study, we propose to project single-omics data onto a unified space by SCCA for data fusion, followed by clustering analysis on the fused data to identify cancer subtypes (Figure 1A). The trained projection matrices, combined with a trained classifier, can be subsequently used to either single-omics or multi-omics classifications (Figure 1B). Using two case studies in ovarian cancer and breast cancer, we demonstrated the usefulness of sparse canonical correlation analysis for cancer classification (SCCA-CC) in cancer classification using multi-omics profiles in the TCGA database^1^ as well as single-omics datasets from other independent datasets. Furthermore, we demonstrated that SCCA-CC is superior to other popular methods such as iCluster in the coherence and clinical relevance of identified cancer subtypes, and the running time consumed.

## Materials and Methods

### Data Collection and Curation

We collected mRNA and miRNA expression profiles for 462 ovarian cancer patients and 451 breast cancer patients from the TCGA database. Single-omics (mRNA or miRNA) datasets were collected from gene expression omnibus (GEO). More specifically, we downloaded one mRNA dataset (Tothill dataset, GSE9891, and *n* = 285) (Tothill et al., 2008), and three miRNA datasets: OC133 (GSE73582, *n* = 133), OC179 (GSE73581, *n* = 179), and Bagnoli (GSE25204, *n* = 130) datasets (Bagnoli et al., 2016) in the ovarian cancer case study. In the breast cancer study, we downloaded the GSE22220 series (Buffa et al., 2011), which includes a mRNA dataset (GSE22219, *n* = 216) and a miRNA dataset (GSE22216, *n* = 210), of which 207 samples have both types of data.

### Penalized Canonical Correlation Analysis and Data Fusion

Canonical correlation analysis was proposed in 1936, which was aimed to use fewer combinatorial variables to reflect the correlation between the original two variable groups (Hotelling, 1936). The measurement of the correlation between the two groups of variables makes it possible to fuse different biometrics. CCA projected the two groups of variables onto a unified space in which the transformed vectors were maximally correlated. As the classical CCA could not handle high-dimensional data with small sample size, sparse CCA introduced convex penalty functions to overcome the challenge (Witten et al., 2009). Given two sets of zero-mean random vectors, *A* = [*a*_1_,*a*_2_,…,*a*_*n*_] ∈ *R*^*D*_*a*_×*n*^, *B* = [*b*_1_,*b*_2_,…,*b*_*n*_] ∈ *R*^*D*_*b*_×*n*^, we can obtain the objective projection matrices *P*_*a*_ ∈ *R*^*D*_*a*_×*m*^ and *P*_*b*_ ∈ *R*^*D*_*b*_×*m*^ corresponding to *A* and *B*, respectively by SCCA to maximize the correlation coefficient ρ:

$$\rho =\frac{P_{a}^{T}\,A\,B^{T}\,P_{b}}{\sqrt{\left(P_{a}^{T}\,A\,A^{T}\,P_{a}\right)\,\left(P_{b}^{T}\,B\,B^{T}\,P_{b}\right)}}$$

Feature-level fusion meant the aggregation of features obtained from various methods of feature extraction. As the features were compressed and extracted to some extent compared with the raw data, the complexity is much lower, and the computation is much more efficient. Much more importantly, feature-level fusion is more tolerant to specific data types, enabling the effective fusion of various omics data. Sparse CCA was implemented using the “CCA” function in R package “PMA”, which performs sparse CCA using the penalized matrix decomposition. Lasso penalty was used to obtain the corresponding canonical vector to enforce sparsity by setting the parameters “typex” and “typez” to “standard”. The sparsity was determined by the penalties applied to the input matrix. The penalties were set to the default value of 0.3 in the analyses. The number of canonical vectors was determined by the lower number of dimensions of the preprocessed mRNA and miRNA data as mentioned in Methods by the parameter “K”. The other parameters were kept by default in the function.

After projecting two types of omics data to the same space, $A^{P}=P_{a}^{T}\,A,A^{P}\in R^{m\times n}$ and $B^{P}=P_{b}^{T}\,B,B^{P}\in R^{m\times n}$, we can subsequently fuse them by a weighted averaging strategy:

$$Z=\alpha \,A^{P}+\left(1-\alpha \right)\,B^{P}=\alpha \,P_{a}^{T}\,A+\left(1-\alpha \right)\,P_{b}^{T}\,B$$

where, α ∈ [0,1] represents the fusion coefficient. In our case studies, we set an equal weight (fusion coefficient) for each type of omics data, and the fused data Z is used for the following consensus clustering analysis.

### Clustering and Classification Analysis

To identify molecular subtypes, we performed unsupervised classification on the fused TCGA data in the unified space. To ensure robustness, we employed the widely adopted consensus clustering method (Monti et al., 2003), with 500 iterations and 0.9 subsampling ratio, to assess the clustering stability. The consensus clustering was implemented by the “ConsensusClusterPlus” function of the R package “ConsensusClusterPlus” with k-means clustering algorithm using Euclidean distance (Monti et al., 2003). The fused TCGA data, together with the subtyping labels, were used to train a classifier. More specifically, we explored various classification methods such as random forests (RF) (R package “randomForest”) (Breiman, 2001), support vector machine (SVM) (R package “e1071”) (Cortes and Vapnik, 1995), k-nearest neighbors algorithm (KNN) (R package “class”) (Venables and Ripley, 2021), minimum distance algorithm (Min-Dis), and Bayesian classifier (R package “e1071”) (Cortes and Vapnik, 1995), and selected the one yielding the lowest error rate for the following analysis. More specifically, in the RF classification analysis of ovarian cancer, the number of trees was set to 1,000 and the other parameters were set by default. In the SVM classification analysis of breast carcinoma (BRCA), we used the radial basis kernel and set the cost of constraints violation to 10.

### Statistical Analysis

Statistical analysis was conducted with R software (version 3.6.1^2^). SigClust (Huang et al., 2015), a statistical method for testing the significance of clustering results, was used to evaluate the subtypes we identified. Differential gene expression analysis was performed by comparing each subtype with the others using the R package “limma” (Ritchie et al., 2015). Biological characterizations of cancer subtypes were based on gene set enrichment analysis (GSEA) using R package “HTSanalyzeR2” (Wang et al., 2011). Cox regression analyses were performed by R package ‘‘survival’’^3^. A *p*- value of less than 0.05 was considered statistically significant in all tests.

## Results

### Molecular Subtyping of Ovarian Cancer Using SCCA-CC

Ovarian cancer is one of the most lethal malignancies in women. Although most ovarian cancer patients can be cured during the early stage, more than 80% of ovarian cancers are diagnosed at advanced stages. Similar to other major malignancies, ovarian cancer has been recognized as a molecularly heterogeneous disease underlying the diverse clinical outcomes. Recently, Tothill et al. (2008) performed unsupervised classification of gene expression profiles for 285 high-grade serous ovarian cancer (HGSOC) samples, resulting in the identification of four distinct subtypes: immunoreactive, differentiated, proliferative, and mesenchymal subgroups. TCGA network recapitulated these subtypes based on transcriptomic profiles of more than 500 OvCa cases (Cancer Genome Atlas Research Network, 2011). More recently, it was found that compared to other subtypes, the mesenchymal subtype displayed higher invasiveness and was associated with poor overall survival (Konecny et al., 2014). Despite the well-established taxonomy, the subtyping studies were based on transcriptomic profiles, ignoring potential heterogeneity at other levels of gene regulations. Furthermore, the classifiers based on gene expression signatures cannot be applied to other types of omics data, greatly limiting the applicability of these classification systems.

#### Unsupervised Classification of the Fused Multi-Omics Data Identified More Clinically Relevant Subtypes

**FIGURE 2:**
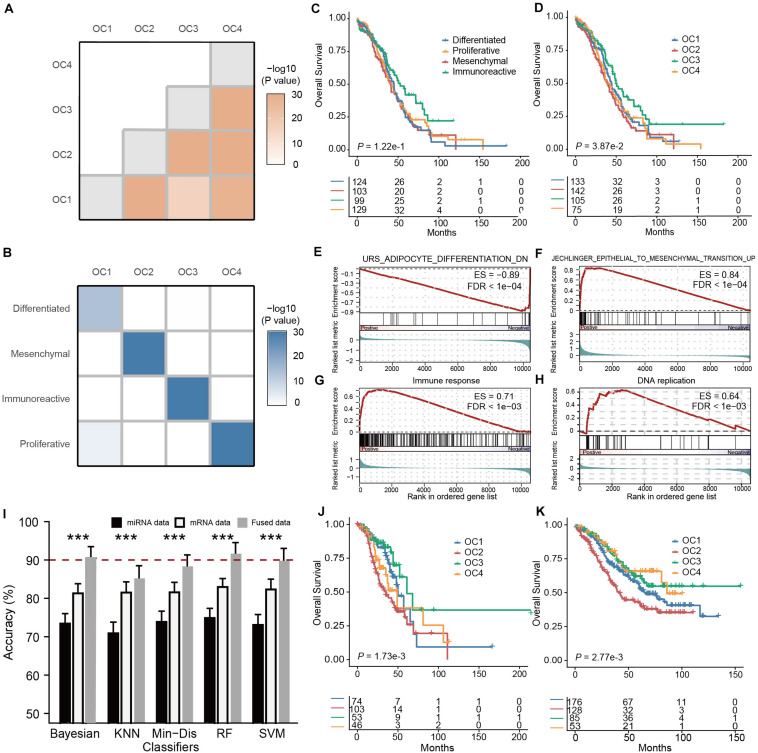
Multi-omics subtyping of ovarian cancer using sparse canonical correlation analysis for cancer classification (SCCA-CC). **(A)** A heatmap showing the statistical significance of the differences between the identified OC subtypes. The color depth is proportionate to the -log_10_(*P-* values) derived from SigClust. **(B)** A heatmap illustrating the association between ovarian cancer subtypes identified by SCCA-CC and the cancer genome atlas (TCGA). The heatmap is colored in proportion to the -log_10_(*P-* values) derived from hypergeometric tests. **(C,D)** Kaplan-Meier plots showing the association of the subtypes identified by panel **(C)** TCGA and **(D)** SCCA-CC, respectively. *P-* values were calculated based on log–rank tests. **(E–H)** GSEA plots illustrating the representative pathways dysregulated in each molecular subtype identified by SCCA-CC. **(I)** A bar plot comparing the classification performance of the five classifiers on mRNA data, miRNA data, and the fused data, respectively. *P-* values were calculated based on Wilcoxon signed–rank tests. *** indicates *P* < 0.001. **(J,K)** Kaplan-Meier plots illustrating the association between the four subtypes identified by SCCA-CC with overall survival in this panel **(J)** the Tothill mRNA dataset and **(K)** the merged miRNA data. *P-* values were calculated based on log–rank tests.

In total, we obtained matched mRNA and miRNA expression profiles from 462 ovarian cancer samples in the TCGA cohort. To eliminate the impacts of magnitude scale and ensure the comparability of data, within each type of omics data we performed z-score normalization and filtered out genes or miRNAs with low between-sample variations (median absolute deviation, or MAD < 0.75). The preprocessed mRNA and miRNA data were subsequently projected onto a unified space using SCCA, followed by data fusion based on a weighted averaging strategy (α = 0.5) (Figure 1A). Using the fused data, we performed consensus clustering and observed that subdivision into four clusters generated the most robust classification (Supplementary Figure 1), suggesting the existence of four major ovarian cancer subtypes (OC1-4). Using SigClust (Huang et al., 2015), a statistical method for testing the significance of clustering results, and we found that indeed the differences between subtypes were statistically significant (all *P* < 0.001, Figure 2A). To interpret the four OC subtypes we identified, we compared our clustering result with the TCGA taxonomy (Cancer Genome Atlas Research Network, 2011; Supplementary Figure 2). Interestingly, we found that each OC subtype identified by SCCA-CC was significantly associated with one of the subtypes identified by TCGA (Figure 2B, all *P* < 0.001, hypergeometric tests; *P* < 0.001, McNemar–Bowker test), suggesting that SCCA-CC recapitulated the four subtypes previously defined, i.e., proliferative, immunoreactive, differentiated, and mesenchymal (Tothill et al., 2008). Notably, the four OC subtypes we identified are significantly associated with overall survival (Figure 2D, *P* = 0.039, log–rank test), while the four TCGA subtypes did not (Figure 2C, *P* = 0.12, log–rank test), supporting our hypothesis that incorporating different types of omics data may identify more clinically relevant subtypes than single-omics approaches.

To further elucidate the OC subtypes, we performed differential gene expression analysis by comparing each subtype with the others and then identified subtype-specific biological functions based on GSEA (Supplementary Table 1). We confirmed that OC1 is differentiated-like, featured with dysregulated cell differentiation signatures; OC2 is mesenchymal-like, displaying upregulated epithelial-to-mesenchymal transition (Jechlinger et al., 2003); OC3 is immunoreactive-like, characterized by activated immune responses; and OC4 is proliferative-like, characterized by upregulated DNA replication, which were all consistent with previous studies (Jechlinger et al., 2003; Verhaak et al., 2013; Wang et al., 2017; Figures 2E–H).

#### The Multi-Omics Classifier Was Able to Classify Both Single-Omics and Multi-Omics Data

A unique advantage of a multi-omics classifier lies in its ability to handle both single-omics data and multi-omics data, making more efficient use of different types of data potentially (Figure 1B). In our study, using the mRNA-miRNA fused data obtained by the projection and fusion from randomly selected 200 TCGA samples, we constructed multiple classifiers based on RF, SVM, KNN, Min-Dis, and Bayesian classifier. Using the clustering labels as the reference, we evaluated the performance of these classifiers on the fused mRNA-miRNA data, the mRNA and the miRNA data alone for the other 262 TCGA samples, respectively. To obtain a stable and robust estimation of the performance, we repeated the tests 100 times. Compared to miRNA-based classification results, all classifiers demonstrated higher accuracies on the mRNA data (Figure 2I, all *P* < 0.001, Wilcoxon Signed–rank tests), and, remarkably, achieved even higher accuracies on the fused data (Figure 2I, all *P* < 0.001, Wilcoxon Signed–rank tests). The results supported our hypothesis that SCCA-CC achieved higher classification performance when more information is incorporated.

#### Independent Validations Verified the General Applicability of Multi-Omics Classification

Among the various classifiers, RF showed relatively higher accuracy and lower volatility (standard deviation): 91.6% using the fused data, 83.0% using only the mRNA data, and 74.8% using only the miRNA data (Figure 2I). Therefore, we trained a multi-omics classifier based on RF using all the 462 TCGA samples. To evaluate the general applicability of the classifier to other independent datasets, we tested the Tothill mRNA dataset (Tothill et al., 2008) (*n* = 279) and a miRNA dataset (*n* = 442) merged from GSE73581, GSE73582, and Bagnoli miRNA (Bagnoli et al., 2015) datasets. In both datasets, the predicted OC subtypes showed a significant association with survival (Figures 2J,K, both *P* < 0.01, log–rank tests). More specifically, patients classified to OC2 (mesenchymal-like) had the worst overall survival, while those classified to OC3 (immunoreactive-like) had the best outcome, which was consistent with previous studies (Jechlinger et al., 2003; Verhaak et al., 2013; Wang et al., 2017). These results demonstrated the multi-omics classifier’s potential to classify other independent datasets with different types of omics data. Notably, it was the first time ever that the three miRNA datasets (GSE73581, GSE73582, and Bagnoli) could be classified, since the previous classification method developed by TCGA only takes mRNA data as input (Cancer Genome Atlas Research Network, 2011).

### Molecular Subtyping of Breast Cancer Using SCCA-CC

Breast carcinoma is the most common type of gynecological cancer, as it alone accounts for 24.2% of all new cancer incidences in women in 2018 (Bray et al., 2018). Over the past two decades, breast cancer mortality has been reduced remarkably since 1989 (Siegel et al., 2020), mainly attributed to population-wide screening based on mammography and improved therapeutics. However, since breast cancer is also a heterogeneous disease, a significant proportion of patients eventually died due to limited benefit from chemotherapy (Jemal et al., 2017). The intrinsic subtypes of breast cancer, including luminal A, luminal B, basal-like, Her2-enriched, and normal-like have been well characterized and widely adopted (Perou et al., 2000; Sorlie et al., 2001). Importantly, the five intrinsic subtypes are characterized by distinct molecular properties, associate with different clinical outcomes. In particular, patients classified to the Her2-positive subtype showed poor survival, while those assigned to the luminal A subtype displayed more favorable outcome (Sorlie et al., 2001, 2003; Yersal and Barutca, 2014; Dai et al., 2015). For breast cancer subtype prediction, PAM50 is the most popular classifier (Parker et al., 2009), but since the classification system was established based on transcriptomic profiles, it cannot be applied to other types of omics data.

#### Unsupervised Classification of the Fused Multi-Omics Data Recapitulated the Five Intrinsic Subtypes of Breast Cancer

**FIGURE 3:**
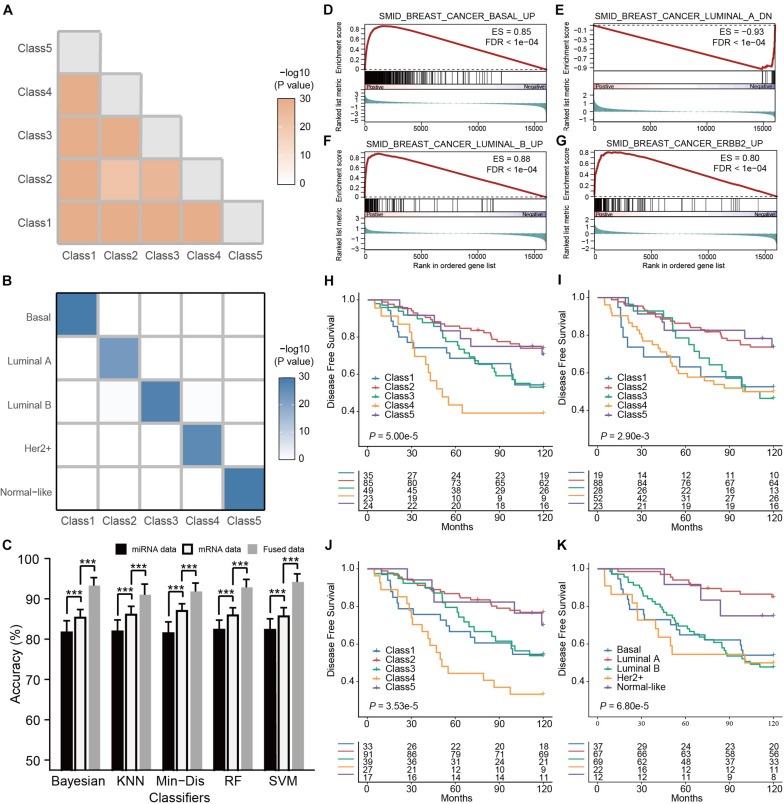
Multi-omics subtyping of breast cancer using sparse canonical correlation analysis for cancer classification (SCCA-CC). **(A)** A heatmap showing the statistical significance of the differences between the identified breast cancer subtypes. The color depth is proportionate to the -log_10_(*P-* values) derived from SigClust. **(B)** A heatmap illustrating the association between the subtypes identified by SCCA-CC and PAM50. The heatmap is colored in proportion to the -log_10_(*P-* values) derived from hypergeometric tests. **(C)** A bar plot comparing the classification performance of the five classifiers on mRNA data, miRNA data and the fused data, respectively. *P-* values were calculated based on Wilcoxon signed–rank tests. *** indicates *P* < 0.001. **(D–G)** GSEA plots illustrating the representative pathways dysregulated in the Class 1 (Basal-like), Class 2 (Luminal A-like), Class 3 (Luminal B-like), and Class 4 (Her2+-like) subtypes identified by SCCA-CC. **(H–J)** Kaplan-Meier plots showing the association of the subtypes identified by SCCA-CC using **(H)** the GSE22219 mRNA dataset, **(I)** the GSE22216 miRNA dataset and **(J)** the fused mRNA and miRNA dataset with disease-free survival. **(K)** Kaplan-Meier plot showing the association of the subtypes identified by PAM50 with disease-free survival. *P-* values were calculated by log–rank tests.

Like our case study in ovarian cancer, we performed unsupervised classification on 451 patient samples of breast cancer with matched mRNA and miRNA expression profiles in the TCGA cohort. Z-score normalization was applied to each type of omics data, followed by the filtering of genes or miRNAs with low between-sample variations (MAD < 0.5). We projected the preprocessed data onto a lower-dimensional space by SCCA for data fusion using the weighted averaging method (α = 0.5) subsequently. Based on consensus clustering of the fused data, we determined the optimal five breast cancer subtypes (Supplementary Figure 3). Pairwise comparisons between the subtypes showed significant differences, suggesting the significance of the clustering (all *P* < 0.001, Figure 3A). Similar to the ovarian cancer study, each breast cancer subtype we identified was significantly associated with an intrinsic subtype classified by PAM50 (Figure 3B and Supplementary Figure 4, all *P* < 0.001, hypergeometric tests; *P* < 0.001, McNemar–Bowker test). To further elucidate the biological properties associated with identified subtypes, we performed differential gene expression analysis by comparing each subtype with the others, followed by GSEA to detect subtype-specific biological functions. The GSEA results (Supplementary Table 1) suggested that Class 1, Class 2, Class 3, and Class 4 were enriched for the gene expression signatures representative of the basal, luminal A, luminal B, and Her2+ (ERBB2) subtypes, respectively (Figures 3D–G; Smid et al., 2008). Since Class 5 recapitulated the normal-like subtype, and therefore we did not notice any particular biological process representative of this subtype.

#### The Multi-Omics Classifier Was Able to Classify Both Single-Omics and Multi-Omics Data

For breast cancer, we also employed the five classification algorithms: RF, SVM, KNN, Min-Dis, and Bayesian classifier to build the classifiers. Using the labels obtained from the consensus clustering as the reference, we randomly selected 200 breast cancer samples from the TCGA cohort to construct classifiers based on the mRNA-miRNA fused data and evaluated the performance on other 251 samples. We repeated the tests 100 times and compared the results of all types of omics data for each classifier. Similar to the ovarian cancer case study, we also found that all the classifiers demonstrated higher accuracies on the mRNA data than on the miRNA data (Figure 3C, all *P* < 0.001, Wilcoxon Signed–rank tests), and they achieved even higher accuracies on the mRNA-miRNA fused data (Figure 3C, all *P* < 0.001, Wilcoxon Signed–rank tests). Consistent with ovarian cancer, our results in breast cancer further demonstrated the improved classification performance of SCCA-CC on multi-omics data.

#### Independent Validations Verified the General Applicability of Multi-Omics Classification

In this case study, SVM demonstrated the best performance and relatively low volatility: 94.8% using the fused data, 88.74% using only the mRNA data, and 80.4% using only the miRNA data. Therefore, we trained a multi-omics classifier based on SVM using all the 451 TCGA samples and evaluated the general applicability of the classifier to other independent datasets. The GSE22220 series, including a mRNA dataset (*n* = 216), a miRNA dataset (*n* = 210), of which 207 samples have both types of data, were used for validations (Buffa et al., 2011). Using either the mRNA or miRNA dataset alone, we found that the predicted subtypes by the multi-omics classifier showed a significant association with survival (Figures 3H,I, both *P* < 0.01, log–rank tests). More interestingly, a higher significance of prognosis was observed using the predicted subtypes based on the fused data (Figure 3J, *P* < 0.001, log–rank test). Regardless of single-omics or multi-omics classifications, the predicted Class 4 (Her2+ like) subtype always displayed the worst overall survival, while the Class 2 (Luminal A like) subtype showed more favorable clinical outcome. These results about clinical associations were consistent with previous studies, demonstrating the general applicability of the multi-omics classifier (Sorlie et al., 2001, 2003; Yersal and Barutca, 2014; Dai et al., 2015). Compared with the PAM50 classification on the same dataset, the SCCA-CC classification was more significantly associated with survival (Figures 3J,K, *P* = 3.5e-5 and 6.8e-5 for SCCA-CC and PAM50 classifications, respectively). Together, our case study suggested that SCCA-CC was able to identify subtypes that are more clinically relevant, and again supported our hypothesis that incorporating different types of omics data may capture more comprehensive intrinsic characteristics of breast cancer than a single data type.

### Benchmark Study

**FIGURE 4:**
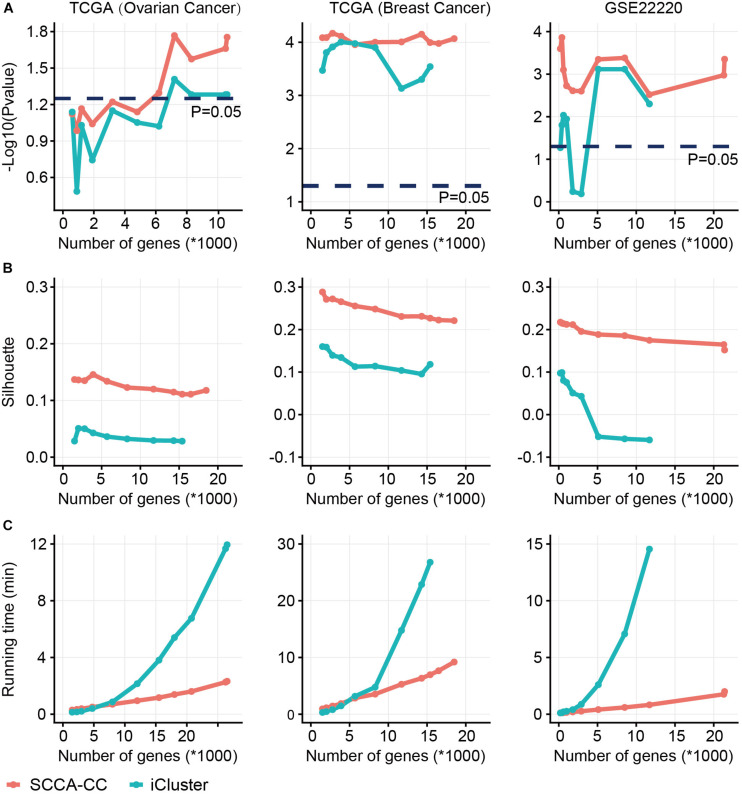
A comparison of sparse canonical correlation analysis for cancer classification (SCCA-CC) with iCluster. Using varying numbers of genes preselected based on MAD, we compared the classification performance between SCCA-CC and iCluster based on **(A)** *P-* values indicative of association with survival calculated by log–rank tests, **(B)** Silhouette score representing the coherence of clusters, and **(C)** the algorithm running time evaluating the computational complexity.

In order to demonstrate the superiority, we directly compared SCCA-CC with iCluster on the datasets we analyzed in the case studies based on three commonly used measures: (i) *P*- values derived from log–rank tests in the Kaplan-Meier analysis to show the association between subtypes and survival; (ii) Silhouette score evaluating the cluster coherence. A higher Silhouette score indicates that samples are more similar within subtypes; and (iii) The algorithm running time evaluating computational complexity. Using varying numbers of genes preselected based on MAD, we performed subtyping analysis using SCCA-CC and iCluster, respectively. As a result, we found SCCA-CC outperformed iCluster based on the three different clustering performance measures in almost all the different scenarios (Figures 4A,B). The algorithm running time is acceptable when a small number of genes were used for both methods, but the time iCluster spent increased exponentially with the number of genes, suggesting better scalability of SCCA-CC (Figure 4C).

To further compare SCCA-CC with other cancer taxonomies and clinical risk factors, we performed Cox regression analyses in ovarian cancer (TCGA dataset) and breast cancer (GSE22220), respectively. For both cancer types, we first employed iCluster to identify the subtypes with default parameters and evaluated the associations between the identified subtypes with the reference subtyping results based on TCGA or PAM50 (Supplementary Figures 5, 6). In ovarian cancer, we found that the SCCA-CC taxonomy showed higher statistical association with patient survival than the classifications based on iCluster and TCGA in the univariate analysis (Table 1). After adjusting for other clinical factors such as age and stage, the SCCA-CC classification did not show significant prognostic power. The lack of significance in the survival difference is not surprising, since the TCGA cohort includes HGSOC patients only, who showed very poor overall survival in general. HGSOC patients are diagnosed at advanced stages, and the 5-year overall survival rate (20–30%) has not significantly improved over the last 20–30 years (Davidson et al., 2014; Konstantinopoulos et al., 2015). These patients were difficult to stratify by other pre-existing classifiers such as the TCGA taxonomy itself (Figure 2C). Even with our SCCA-CC classifier, the survival difference is marginally significant (*P* = 0.031, Univariate Cox regression in Table 1; *P* = 0.0387, and log–rank test in Figure 2D) in the TCGA cohort. However, the independent validation dataset, with only miRNA expression profiles for ovarian cancer, includes not only high-grade serous tumors but also other ovarian cancer histotypes that are less aggressive. Therefore, in the miRNA cohort, the overall survival of the patients showed much higher diversity (Figures 2J,K). Based on the univariate and multivariate analysis, we found the SCCA-CC classification showed significant prognostic power, even after adjusting for age and stage information (Supplementary Table 3). Furthermore, in breast cancer SCCA-CC also outperformed iCluster classification and the PAM classification, and the prognostic power remains after adjusting age and grade factors (Table 2). Together, using both the ovarian and breast cancer case studies, we demonstrated the better performance of SCCA-CC in identifying molecular subtypes that are more coherent and clinically relevant.

**TABLE 1:** Univariate and multivariate Cox regression analyses in ovarian cancer using the TCGA dataset.

	Univariate		Multivariate	
	HR (95% CI)	*P-* value	HR (95% CI)	*P-* value
Age (≥65 vs. < 65)	1.37 (1.07–1.76)	0.014	1.32 (1.02–1.71)	0.034
Stage (Late vs. Early)	2.33 (1.10∼4.94)	0.028	2.21 (1.04–4.70)	0.039
SCCA-CC (multinomial)	1.04 (0.85–1.08)	0.466		
TCGA labels (multinomial)	0.94 (0.95–1.19)	0.279		
SCCA-CC (OC2 vs. OC1/3/4)	1.33 (1.03∼1.73)	0.031	1.21 (0.93–1.58)	0.161
iCluster (iCluster 1 vs. iCluster 2–4)	1.25 (0.94∼1.67)	0.12		
TCGA labels (Mesenchymal vs. Others)	0.82 (0.91∼1.63)	0.18		
				

**TABLE 2:** Univariate and multivariate Cox regression analyses in breast cancer using the GSE22220 series dataset.

	Univariate		Multivariate	
	HR (95% CI)	*P-* value	HR (95% CI)	*P-* value
Age (≥65 vs. < 65)	2.28 (1.42∼3.68)	0.0007	1.81 (1.09–2.99)	0.021
Grade (2–3 vs. 1)	1.82 (1.06∼3.11)	0.030	1.55 (0.89–2.68)	0.118
ER status (1 vs. 0)	0.80 (0.51∼1.26)	0.33		
SCCA-CC (multinomial)	0.86 (0.96–1.39)	0.127		
PAM50 (multinomial)	1.06 (0.78–1.15)	0.575		
SCCA-CC (Class 4 vs. Classes 1–3, 5)	2.95 (1.73∼5.02)	< 0.0001	1.95 (1.07–3.55)	0.030
iCluster (iCluster 5 vs. iCluster 1–4)	2.49 (1.54∼4.02)	0.0002	1.68 (0.97–2.91)	0.063
PAM50 (Her2+ vs. others)	1.77 (0.93∼3.35)	0.08		

### Interpretations of the Canonical Variate Pairs

Sparse CCA provides sets of variables with sparse loadings, which is consistent with the belief that only a small number of genes are expressed under specific conditions (Parkhomenko et al., 2007). Previous studies have used sparse CCA to investigate the associations between different types of omics data, e.g., identification of sets of genes that are correlated with sets of SNPs and copy number variations (Parkhomenko et al., 2007, 2009; Waaijenborg et al., 2008). For better understanding the biology underlying the CCA we further analyzed the pairwise correlations of mRNAs and miRNAs, and build miRNA-mRNA regulatory networks in our case studies.

**FIGURE 5:**
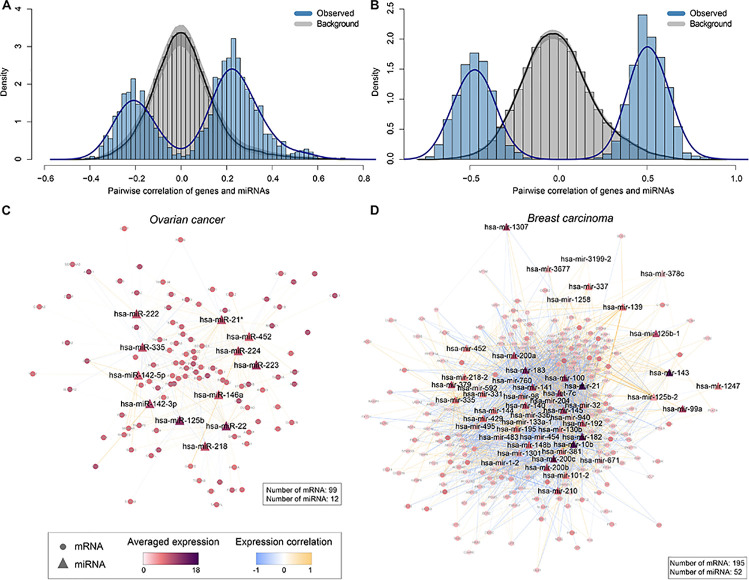
Interpretations of the canonical variate pairs. **(A,B)** The distributions of observed pairwise correlation coefficients between non-zero mRNA variables and miRNA variables in the first canonical variates, compared to the corresponding background distributions in this panel **(A)** ovarian cancer and **(B)** breast carcinoma (BRCA), respectively. The gray area represents 95% confidence intervals. **(C,D)** MiRNA-mRNA regulatory networks constructed based on non-zero mRNA and miRNA variables selected by sparse CCA in this panel **(C)** ovarian cancer and **(D)** breast carcinoma, respectively. Triangles and circles represent miRNAs and mRNAs, respectively. Edges represent the interactions between miRNAs and mRNAs experimentally validated and/or predicted by databases including miRecords, miRTarBase, TarBase, TargetScan, PITA, and miRanda. Nodes are colored in proportion to the averaged log2 transformed expression levels of mRNAs/miRNAs across all TCGA samples, and edges are colored based on Pearson correlation coefficients between the expression levels of miRNAs and mRNAs.

In ovarian cancer, we checked the first canonical variate pair of mRNAs and miRNAs and found 105 non-zero mRNA variables and 12 non-zero miRNA variables. Pairwise correlation coefficients (*n* = 1260) were calculated between these variables using their original expression data. Interestingly, we found apparent correlation (negative or positive) between the expression levels of mRNAs and miRNAs, suggesting their potential interactions (Figure 5A). As a comparison, we generated a background distribution of correlation coefficients based on random sampling of 1260 pairs of mRNAs and miRNAs from all the input data, repeating for 1,000 times. As a result, the randomly selected mRNAs and miRNAs showed lack of association (Figure 5A), suggesting the functional relevance of the non-zero mRNAs and miRNAs variables. Based on the interesting correlation observed, we hypothesize that physical interactions may underlie the expression associations between these mRNAs and miRNAs selected by sparse CCA. To test the hypothesis, we built a miRNA-mRNA regulatory network by collecting both experimentally validated miRNA-target interactions (from miRecords (Xiao et al., 2009), miRTarBase (Huang et al., 2020), and TarBase (Karagkouni et al., 2018) and predicted miRNA-target interactions with evolutionary conservation (from TargetScan (Agarwal et al., 2015), PITA (Kertesz et al., 2007) and miRanda (Enright et al., 2003). As expected, most of the mRNAs (99 out of the total 105) and all the miRNAs are interconnected (Figure 5C), suggesting that these miRNAs have intense physical interactions with the mRNAs. As an example, CDR2L, with the second largest weight, is the target of hsa-miR-125b, hsa-miR-142-3p, hsa-miR-142-5p and hsa-miR-222 based on targetScan, and/or PITA predictions (Kertesz et al., 2007; Agarwal et al., 2015; Supplementary Table 2). Similarly, MMD, with the third largest weight, is the target of hsa-miR-142-3p, hsa-miR-142-5p, hsa-miR-223, hsa-miR-224, and hsa-miR-335 (Kertesz et al., 2007; Agarwal et al., 2015; Supplementary Table 2).

Similarly, in BRCA we checked the first canonical variate pair of genes and miRNAs dataset and found 204 non-zero mRNA variables and 57 non-zero miRNA variables. Pairwise correlation coefficients (*n* = 11628) were calculated between these variables using their original expression data. As a result, we also found strong correlation (negative or positive) between the expression levels of mRNAs and miRNAs (Figure 5B). A background distribution of correlation coefficients based on random sampling of 11,628 pairs of mRNAs and miRNAs from all the input data, repeating for 1,000 times. As expected, the randomly selected mRNAs and miRNAs also showed lack of association (Figure 5B). We further built a miRNA-mRNA regulatory network to investigate whether physical interactions may also underlie the expression associations of mRNAs and miRNAs selected by sparse CCA in BRCA. Indeed, most of the genes (195 out of the total 204) and the miRNAs (52 out of the total 57) are interconnected (Figure 5D), suggesting that these miRNAs have intense physical interactions with the mRNAs. For instance, Gene UBE2T with the highest weight is the target of hsa-mir-96, hsa-mir-200c, has-miR-182 based on targetScan, and/or PITA predictions (Kertesz et al., 2007; Agarwal et al., 2015; Supplementary Table 2). Gene CKS2, with the second highest weight, is the target of hsa-mir-200c, has-miR-429, and has-miR-33b (Kertesz et al., 2007; Agarwal et al., 2015; Supplementary Table 2).

Together, these results provide compelling evidence that the sparse CCA selected biologically relevant genes and miRNAs, which explains their strong expression correlation enabling multi-omic data fusion in the projected space.

## Discussion

Cancer molecular heterogeneity hampers the selection of patients for more optimized clinical management and the design of targeted agents. During the last decade, tremendous efforts have been made to dissecting the inter-tumor heterogeneity in an overwhelming number of studies based on unsupervised classification of high-throughput omics profiles. These studies gained novel insights into cancer biology with important clinical implications, which laid a solid foundation for precision medicine. However, most of these studies were based on single-omics data, especially transcriptomic data, which ignored other genetic and epigenetic levels of gene regulation, and resulting in only partial understanding of cancer heterogeneity. Recent studies have seen a growing interest in integrating multiple types of omics data for more comprehensive cancer subtyping, but few existing methods can classify both single-omics and multi-omics data. In this study, we developed SCCA-CC, a robust and efficient framework for cancer subtyping and classifications based on data fusion using sparse CCA followed by unsupervised classification. Using two case studies on multiple independent cohorts, we demonstrated that SCCA-CC was able to identify biologically meaningful and clinically more relevant taxonomies.

Conventional CCA may suffered from the high dimensionality of genomic data where the number of observations greatly exceeds the number of samples, leading to high risk of potential collinearity and unstable estimates (Waaijenborg et al., 2008; Parkhomenko et al., 2009; Boutte and Liu, 2010). PCA is a powerful dimension reduction method, which has been used prior to CCA in some applications. However, in our study, we did not perform PCA prior to CCA due to the following considerations: (1) We employed sparse CCA but not the conventional CCA in our study. In the sparse CCA (Witten et al., 2009), a penalized matrix decomposition is introduced using a LASSO penalty to compute a rank-K approximation of a matrix (Witten et al., 2009; Lin et al., 2013). This is inspired by several penalization methods presented in the regression context (Zou et al., 2006; Wright et al., 2009; Wu et al., 2009). As reported before, the problem of multicollinearity can be mitigated by the use of sparse loadings in the CCA algorithm (Waaijenborg and Zwinderman, 2009; Witten et al., 2009; Boutte and Liu, 2010; Lin et al., 2013). (2) Cross-platform applicability. In practice, the strategy to perform PCA before CCA may be difficult to be applied to other datasets based on different gene expression profiling platforms. For instance, a lot of newly identified genes in RNA-seq data were often missing in early gene expression microarrays published many years ago. (3) After performing PCA prior to CCA, the generalized eigenvector problem is changed into the eigen-system computation of a nonsymmetric matrix which is unstable as previously reported (Swets and Weng, 1996). (4) Performing PCA prior to CCA may discard dimensions that contain important discriminative information (Xing et al., 2016).

For both ovarian and breast cancers, the subtypes identified by SCCA-CC recapitulated the taxonomies previously established, as suggested by the pairwise statistical tests and GSEA. However, SCCA-CC derived subtypes showed a more significant association with clinical outcomes. Notably, ovarian cancer subtypes identified by SCCA-CC were significantly associated with overall survival in the TCGA cohort, while the four subtypes previously defined by TCGA did not show any significant clinical association. On independent mRNA, miRNA, and fused datasets, SCCA-CC demonstrated consistent clinical associations in both our ovarian and breast cancer studies. These results demonstrated that SCCA-CC is able to detect the biologically coherent subgroups in different types of single-omics data, and incorporating multiple types of omics data can further improve the prediction performance.

More importantly, a number of published studies with only miRNA expression profiles cannot be classified using existing subtyping systems based on mRNA expression signatures. SCCA-CC presented a unique advantage in its ability to classify both single-omics data and multi-omics data, which significantly extends the general applicability to make efficient use of public resources. More specifically, we constructed multi-omics classifiers using the fused data with the consensus clustering labels as the reference and evaluated the robustness of the classification performance by iterative testing. The validity of multi-omics classifiers was verified by the observed significant prognostic power on both the mRNA dataset and the miRNA dataset. Notably, it was the first time ever that the miRNA datasets could be classified since the previous classification assays only take mRNA data as input.

In a benchmark study against iCluster, SCCA-CC also demonstrated its superiority in the higher coherence and clinical relevance of identified cancer subtypes, and lower computational complexity. Furthermore, the strength of SCCA-CC also lies in the biological interpretability. The non-zero mRNAs and miRNAs selected by sparse CCA had strong correlation in their expression levels, which can be explained by their intense physical interactions. These results provide compelling evidence that the sparse CCA selected biologically relevant genes and miRNAs.

Despite the demonstrated usefulness, the major limitation of SCCA-CC lies in the limited types of omics data we used in the study. Only mRNA and miRNA data were fused for classification, likely missing heterogeneity occurring at other omics levels. Thus, our future work will focus on integrating more types of omics data to dissect the heterogeneity more comprehensively. Considering the differences in the dimensionalities and data scales between various types of omics data, how to properly preprocess the data for effective data fusion remains a significant challenge.

## Data Availability Statement

The original contributions presented in the study are included in the article/Supplementary Material, further inquiries can be directed to the corresponding author.

## Author Contributions

XW contributed to study concept and design. LQ, WW, and TW contributed to data collection, analysis, and interpretation. XW contributed to critical revision of the manuscript for important intellectual content. LZ and LH provided important advice and assistance for manuscript drafting. XW supervised the study. All authors read and approved the final manuscript.

## Conflict of Interest

The authors declare that the research was conducted in the absence of any commercial or financial relationships that could be construed as a potential conflict of interest.

## Footnotes

1 https://cancergenome.nih.gov/

2 http://www.Rproject.org

3 https://github.com/therneau/survival

## Supplementary Material

Supplementary Figure 1 Consensus clustering based on the fused data in ovarian cancer. **(A)** Heatmap illustrating the consensus matrices for *k* = 4, 5, and 6. **(B)** Consensus cumulative distribution function (CDF) plot for k varying from 2 to 6. **(C)** Delta area plot shows the relative change in the area under the consensus cumulative distribution function (CDF) curve comparing k and k–1. At *k* = 4, there is no appreciable increase (Delta area < 0.1). **(D)** Gap statistic suggesting the optimal number of clusters is four in the TCGA dataset. Error bars indicate SEM.

Supplementary Figure 2 A heatmap illustrating the pairwise comparison between the subtypes identified by SCCA-CC and those defined by the cancer genome atlas (TCGA) in the TCGA ovarian cancer dataset.

Supplementary Figure 3 Consensus clustering based on the fused data in breast cancer. **(A)** Heatmap illustrating the consensus matrices for *k* = 5, 6, and 7. **(B)** Consensus cumulative distribution function (CDF) plot for k varying from 2 to 7. **(C)** Delta area plot shows the relative change in the area under the consensus cumulative distribution function (CDF) curve comparing k and k–1. At *k* = 5, there is no appreciable increase (Delta area < 0.1). **(D)** Gap statistic suggesting the optimal number of clusters is five in the TCGA dataset. Error bars indicate SEM.

Supplementary Figure 4 A heatmap illustrating the pairwise comparison between the subtypes identified by SCCA-CC and those defined by PAM50 in the TCGA breast cancer dataset.

Supplementary Figure 5 Heatmaps showing the pairwise comparison between the subtypes identified by iCluster and the cancer genome atlas (TCGA) in the TCGA ovarian cancer dataset. **(A)** The statistical significance of association quantified by hypergeometric tests. **(B)** The detailed confusion matrix.

Supplementary Figure 6 Heatmaps showing the pairwise comparison between the subtypes identified by iCluster and PAM50 in the GSE22219 breast cancer dataset. **(A)** The statistical significance of association quantified by hypergeometric tests. **(B)** The detailed confusion matrix.

Supplementary Table 1 The result of gene set enrichment analysis (xlsx).

Supplementary Table 2 The weights of canonical variates (xlsx).

Supplementary Table 3 Univariate and multivariate Cox regression analyses in the independent miRNA validation datasets (xlsx).

## References

[B1] Agarwal V. Bell G. W. Nam J.-W. Bartel D. P. (2015). Predicting effective microRNA target sites in mammalian mRNAs. *Elife* 4:e05005. 10.7554/eLife.05005 26267216 PMC4532895

[B2] Bagnoli M. Canevari S. Califano D. Losito S. Maio M. D. Raspagliesi F. (2016). Development and validation of a microRNA-based signature (MiROvaR) to predict early relapse or progression of epithelial ovarian cancer: a cohort study. *Lancet Oncol.* 17 1137–1146. 10.1016/s1470-2045(16)30108-5 27402147

[B3] Bagnoli M. De Cecco L. Granata A. Nicoletti R. Marchesi E. Alberti P. (2015). Identification of a chrXq27.3 microRNA cluster associated with early relapse in advanced stage ovarian cancer patients. *Oncotarget* 6:9643. 10.18632/oncotarget.3998 26002433 PMC4496384

[B4] Bersanelli M. Mosca E. Remondini D. Giampieri E. Sala C. Castellani G. (2016). Methods for the integration of multi-omics data: mathematical aspects. *BMC Bioinform.* 17 (Suppl. 2):15. 10.1186/s12859-015-0857-9 PMC4959355 26821531

[B5] Boutte D. Liu J. (2010). “Sparse canonical correlation analysis applied to fMRI and genetic data fusion”, in *Proceedings of the 2010 IEEE International Conference on Bioinformatics and Biomedicine (BIBM)*, Hong Kong, 422–426. 10.1109/BIBM.2010.5706603 PMC6353614 30713779

[B6] Bray F. Ferlay J. Soerjomataram I. Siegel R. L. Torre L. A. Jemal A. (2018). Global cancer statistics 2018: GLOBOCAN estimates of incidence and mortality worldwide for 36 cancers in 185 countries. *CA Cancer J. Clin.* 68 394–424. 10.3322/caac.21492 30207593

[B7] Breiman L. (2001). Random forests. *Mach. Learn.* 45 5–32.

[B8] Buffa F. M. Camps C. Winchester L. Snell C. E. Gee H. E. Sheldon H. (2011). microRNA-associated progression pathways and potential therapeutic targets identified by integrated mRNA and microRNA expression profiling in breast cancer. *Cancer Res.* 71 5635–5645. 10.1158/0008-5472.can-11-0489 21737487

[B9] Cancer Genome Atlas Research Network (2011). Integrated genomic analyses of ovarian carcinoma. *Nature* 474 609–615. 10.1038/nature10166 21720365 PMC3163504

[B10] Cortes C. Vapnik V. (1995). Support-vector networks. *Mach. Learn.* 20 273–297.

[B11] Dai X. Li T. Bai Z. Yang Y. Liu X. Zhan J. (2015). Breast cancer intrinsic subtype classification, clinical use and future trends. *Am. J. Cancer Res.* 5 2929–2943. 26693050 PMC4656721

[B12] Davidson B. Rosenfeld Y. B. Z. Holth A. Hellesylt E. Tropé C. G. Reich R. (2014). VICKZ2 protein expression in ovarian serous carcinoma effusions is associated with poor survival. *Hum. Pathol.* 45 1520–1528. 10.1016/j.humpath.2014.03.005 24814803

[B13] Enright A. J. John B. Gaul U. Tuschl T. Sander C. Marks D. S. (2003). MicroRNA targets in *Drosophila*. *Genome Biol.* 5:R1. 14709173 10.1186/gb-2003-5-1-r1 PMC395733

[B14] Hotelling H. (1936). Relations between two sets of variates. *Biometrika* 28:321. 10.2307/2333955

[B15] Huang H. Liu Y. Yuan M. Marron J. S. (2015). Statistical significance of clustering using Soft thresholding. *J. Comput. Graph. Stat.* 24 975–993. 10.1080/10618600.2014.948179 26755893 PMC4706235

[B16] Huang H.-Y. Lin Y.-C.-D. Li J. Huang K.-Y. Shrestha S. Hong H.-C. (2020). miRTarBase 2020: updates to the experimentally validated microRNA-target interaction database. *Nucleic Acids Res.* 48 D148–D154. 31647101 10.1093/nar/gkz896 PMC7145596

[B17] Jechlinger M. Grunert S. Tamir I. H. Janda E. Lüdemann S. Waerner T. (2003). Expression profiling of epithelial plasticity in tumor progression. *Oncogene* 22 7155–7169. 10.1038/sj.onc.1206887 14562044

[B18] Jemal A. Ward E. M. Johnson C. J. Cronin K. A. Ma J. Ryerson B. (2017). Annual report to the nation on the status of cancer, 1975-2014, featuring survival. *J. Natl. Cancer Inst.* 109:djx030. 10.1093/jnci/djx030 28376154 PMC5409140

[B19] Karagkouni D. Paraskevopoulou M. D. Chatzopoulos S. Vlachos I. S. Tastsoglou S. Kanellos I. (2018). DIANA-TarBase v8: a decade-long collection of experimentally supported miRNA-gene interactions. *Nucleic Acids Res.* 46 D239–D245. 29156006 10.1093/nar/gkx1141 PMC5753203

[B20] Kertesz M. Iovino N. Unnerstall U. Gaul U. Segal E. (2007). The role of site accessibility in microRNA target recognition. *Nat. Genet.* 39 1278–1284. 10.1038/ng2135 17893677

[B21] Kirk P. Griffin J. E. Savage R. S. Ghahramani Z. Wild D. L. (2012). Bayesian correlated clustering to integrate multiple datasets. *Bioinformatics* 28 3290–3297. 10.1093/bioinformatics/bts595 23047558 PMC3519452

[B22] Konecny G. E. Wang C. Hamidi H. Winterhoff B. Kalli K. R. Dering J. (2014). Prognostic and therapeutic relevance of molecular subtypes in high-grade serous ovarian cancer. *J. Natl. Cancer Inst.* 106:dju249. 10.1093/jnci/dju249 25269487 PMC4271115

[B23] Konstantinopoulos P. A. Ceccaldi R. Shapiro G. I. D’Andrea A. D. (2015). Homologous recombination deficiency: exploiting the fundamental vulnerability of ovarian cancer. *Cancer Discov.* 5 1137–1154. 10.1158/2159-8290.cd-15-0714 26463832 PMC4631624

[B24] Lin D. Zhang J. Li J. Calhoun V. D. Deng H.-W. Wang Y.-P. (2013). Group sparse canonical correlation analysis for genomic data integration. *BMC Bioinform.* 14:245. 10.1186/1471-2105-14-245 23937249 PMC3751310

[B25] Lock E. F. Hoadley K. A. Marron J. S. Nobel A. B. (2013). Joint and individual variation explained (JIVE) for integrated analysis of multiple data types. *Ann Appl Stat.* 7 523–542. 10.1214/12-AOAS597 23745156 PMC3671601

[B26] Monti S. Tamayo P. Mesirov J. Golub T. (2003). Consensus clustering: a resampling-based method for class discovery and visualization of gene expression microarray data. *Mach. Learn.* 52:118.

[B27] Parker J. S. Mullins M. Cheang M. C. U. Leung S. Voduc D. Vickery T. (2009). Supervised risk predictor of breast cancer based on intrinsic subtypes. *J. Clin. Oncol.* 27 1160–1167. 10.1200/jco.2008.18.1370 19204204 PMC2667820

[B28] Parkhomenko E. Tritchler D. Beyene J. (2007). Genome-wide sparse canonical correlation of gene expression with genotypes. *BMC Proc.* 1 (Suppl. 1):S119. 18466460 10.1186/1753-6561-1-s1-s119 PMC2367499

[B29] Parkhomenko E. Tritchler D. Beyene J. (2009). Sparse canonical correlation analysis with application to genomic data integration. *Stat. Appl. Genet. Mol. Biol.* 8:1. 10.2202/1544-6115.1406 19222376

[B30] Perou C. M. Sørlie T. Eisen M. B. van de Rijn M. Jeffrey S. S. Rees C. A. (2000). Molecular portraits of human breast tumours. *Nature* 406 747–752. 10963602 10.1038/35021093

[B31] Ritchie M. E. Phipson B. Wu D. Hu Y. Law C. W. Shi W. (2015). limma powers differential expression analyses for RNA-sequencing and microarray studies. *Nucleic Acids Res.* 43:e47. 10.1093/nar/gkv007 25605792 PMC4402510

[B32] Shen R. Olshen A. B. Ladanyi M. (2009). Integrative clustering of multiple genomic data types using a joint latent variable model with application to breast and lung cancer subtype analysis. *Bioinformatics* 25 2906–2912. 10.1093/bioinformatics/btp543 19759197 PMC2800366

[B33] Siegel R. L. Miller K. D. Jemal A. (2020). Cancer statistics, 2020. *CA Cancer J. Clin.* 70 7–30. 31912902 10.3322/caac.21590

[B34] Smid M. Wang Y. Zhang Y. Sieuwerts A. M. Yu J. Klijn J. G. M. (2008). Subtypes of breast cancer show preferential site of relapse. *Cancer Res.* 68 3108–3114. 10.1158/0008-5472.can-07-5644 18451135

[B35] Sorlie T. Perou C. M. Tibshirani R. Aas T. Geisler S. Johnsen H. (2001). Gene expression patterns of breast carcinomas distinguish tumor subclasses with clinical implications. *Proc. Natl. Acad. Sci. U.S.A.* 98 10869–10874. 10.1073/pnas.191367098 11553815 PMC58566

[B36] Sorlie T. Tibshirani R. Parker J. Hastie T. Marron J. S. Nobel A. (2003). Repeated observation of breast tumor subtypes in independent gene expression data sets. *Proc. Natl. Acad. Sci. U.S.A.* 100 8418–8423. 10.1073/pnas.0932692100 12829800 PMC166244

[B37] Swets D. L. Weng J. J. (1996). Using discriminant eigenfeatures for image retrieval. *IEEE Transact. Patt. Anal. Mach. Intel.* 18 831–836. 10.1109/34.531802

[B38] Tothill R. W. Tinker A. V. George J. Brown R. Fox S. B. Lade S. (2008). Novel molecular subtypes of serous and endometrioid ovarian cancer linked to clinical outcome. *Clin. Cancer Res.* 14 5198–5208. 10.1158/1078-0432.ccr-08-0196 18698038

[B39] Venables W. N. Ripley B. D. (2021). *Modern Applied Statistics With S*, 4th Edn. Available online at: https://www.stats.ox.ac.uk/pub/MASS4/ (accessed June 23, 2021).

[B40] Verhaak R. G. W. Tamayo P. Yang J.-Y. Hubbard D. Zhang H. Creighton C. J. (2013). Prognostically relevant gene signatures of high-grade serous ovarian carcinoma. *J. Clin. Invest.* 123 517–525. 23257362 10.1172/JCI65833 PMC3533304

[B41] Waaijenborg S. Zwinderman A. H. (2009). Sparse canonical correlation analysis for identifying, connecting and completing gene-expression networks. *BMC Bioinform.* 10:315. 10.1186/1471-2105-10-315 PMC2760886 19785734

[B42] Waaijenborg S. Verselewel de Witt Hamer P. C. Zwinderman A. H. (2008). Quantifying the association between gene expressions and DNA-markers by penalized canonical correlation analysis. *Stat. Appl. Genet. Mol. Biol.* 7:3. 10.2202/1544-6115.1329 18241193

[B43] Wang B. Mezlini A. M. Demir F. Fiume M. Tu Z. Brudno M. (2014). Similarity network fusion for aggregating data types on a genomic scale. *Nat. Methods* 11 333–337. 10.1038/nmeth.2810 24464287

[B44] Wang C. Armasu S. M. Kalli K. R. Maurer M. J. Heinzen E. P. Keeney G. L. (2017). Pooled clustering of high-grade serous ovarian cancer gene expression leads to novel consensus subtypes associated with survival and surgical outcomes. *Clin. Cancer Res.* 23 4077–4085. 10.1158/1078-0432.ccr-17-0246 28280090 PMC5567822

[B45] Wang X. Terfve C. Rose J. C. Markowetz F. (2011). HTSanalyzeR: an R/Bioconductor package for integrated network analysis of high-throughput screens. *Bioinformatics* 27 879–880. 10.1093/bioinformatics/btr028 21258062 PMC3051329

[B46] Witten D. M. Tibshirani R. Hastie T. (2009). A penalized matrix decomposition, with applications to sparse principal components and canonical correlation analysis. *Biostatistics* 10 515–534. 10.1093/biostatistics/kxp008 19377034 PMC2697346

[B47] Wright J. Yang A. Y. Ganesh A. Sastry S. S. Ma Y. (2009). Robust face recognition via sparse representation. *IEEE Trans. Pattern Anal. Mach. Intell.* 31 210–227. 19110489 10.1109/TPAMI.2008.79

[B48] Wu T. T. Chen Y. F. Hastie T. Sobel E. Lange K. (2009). Genome-wide association analysis by lasso penalized logistic regression. *Bioinformatics* 25 714–721. 10.1093/bioinformatics/btp041 19176549 PMC2732298

[B49] Xiao F. Zuo Z. Cai G. Kang S. Gao X. Li T. (2009). miRecords: an integrated resource for microRNA-target interactions. *Nucleic Acids Res.* 37 D105–D110. 18996891 10.1093/nar/gkn851 PMC2686554

[B50] Xing X. Wang K. Yan T. Lv Z. (2016). Complete canonical correlation analysis with application to multi-view gait recognition. *Pattern Recogn.* 50 107–117. 10.1016/j.patcog.2015.08.011

[B51] Yersal O. Barutca S. (2014). Biological subtypes of breast cancer: Prognostic and therapeutic implications. *World J. Clin. Oncol.* 5 412–424. 10.5306/wjco.v5.i3.412 25114856 PMC4127612

[B52] Zhang S. Li Q. Liu J. Zhou X. J. (2011). A novel computational framework for simultaneous integration of multiple types of genomic data to identify microRNA-gene regulatory modules. *Bioinformatics* 27 i401–i409. 21685098 10.1093/bioinformatics/btr206 PMC3117336

[B53] Zhang S. Liu C.-C. Li W. Shen H. Laird P. W. Zhou X. J. (2012). Discovery of multi-dimensional modules by integrative analysis of cancer genomic data. *Nucleic Acids Res.* 40 9379–9391. 10.1093/nar/gks725 22879375 PMC3479191

[B54] Zhao L. Lee V. H. F. Ng M. K. Yan H. Bijlsma M. F. (2019). Molecular subtyping of cancer: current status and moving toward clinical applications. *Brief. Bioinform.* 20 572–584. 10.1093/bib/bby026 29659698

[B55] Zou H. Hastie T. Tibshirani R. (2006). Sparse principal component analysis. *J. Comput. Graph. Stat.* 15 265–286.

