# Supplementary material for: Multi-Omics Data Fusion for Cancer Molecular Subtyping Using Sparse Canonical Correlation Analysis
Source: Front Genet. 2021 Jul 22;12:607817. doi: 10.3389/fgene.2021.607817 (PMC8341864; doi:10.3389/fgene.2021.607817)
Supplement: Supplementary Figure 1 — Consensus clustering based on the fused data in ovarian cancer. (A) Heatmap illustrating the consensus matrices for k = 4, 5, and 6. (B) Consensus cumulative distribution function (CDF) plot for k varying from 2 to 6. (C) Delta area plot shows the relative change in the area under the consensus cumulative distribution function (CDF) curve comparing k and k–1. At k = 4, there is no appreciable increase (Delta area < 0.1). (D) Gap statistic suggesting the optimal number of clusters is four in the TCGA dataset. Error bars indicate SEM. [file Data_Sheet_1.PDF]

Supplementary Material

1 Supplementary Figures

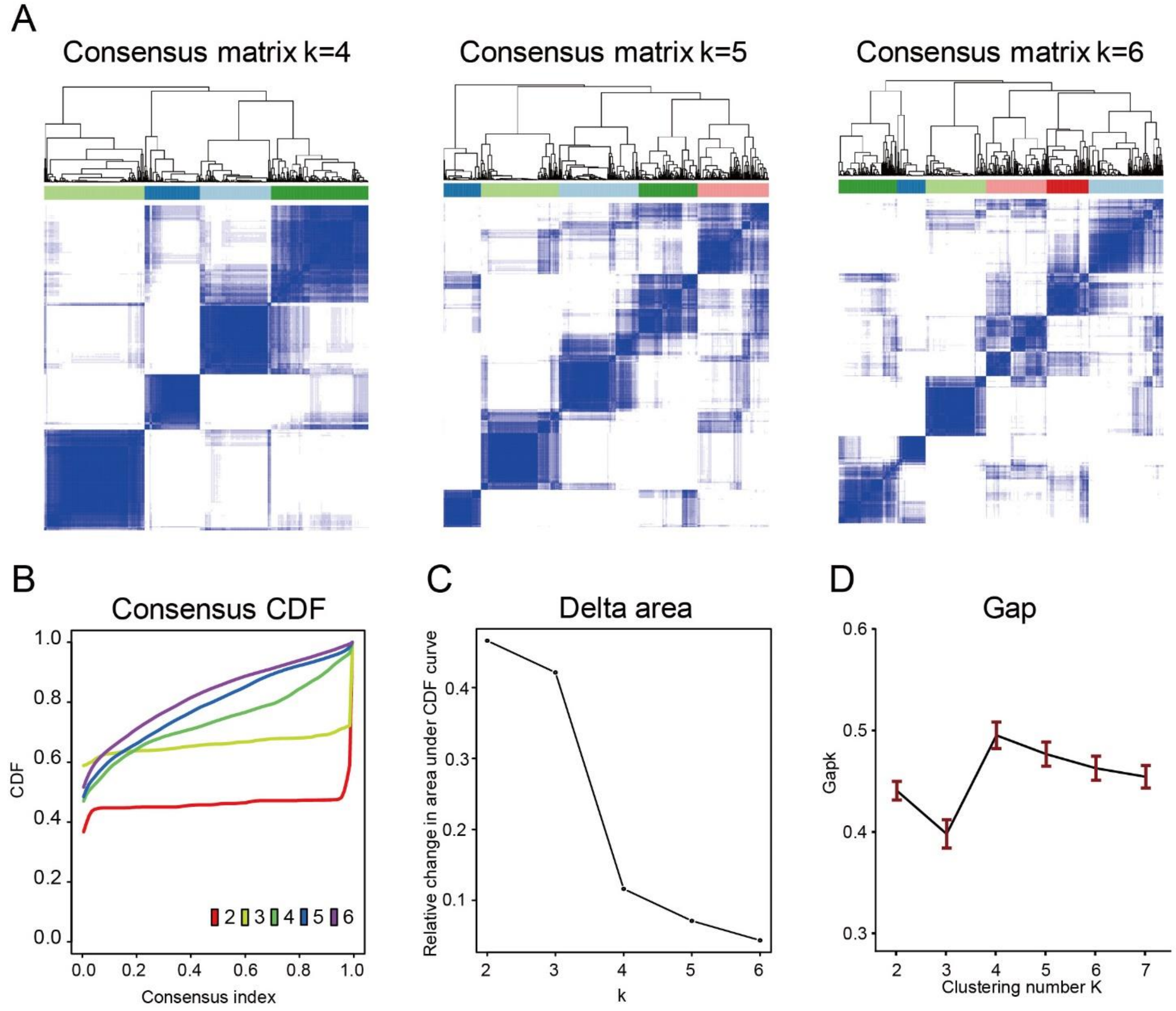

Supplementary Figure 1. Consensus clustering based on the fused data in ovarian cancer. **(A)** Heatmap illustrating the consensus matrices for k = 4, 5 and 6. **(B)** Consensus cumulative distribution function (CDF) plot for k varying from 2 to 6. **(C)** Delta area plot shows the relative change in the area under the consensus cumulative distribution function (CDF) curve comparing k and k – 1. At k = 4, there is no appreciable increase (Delta area < 0.1). **(D)** Gap statistic suggesting the optimal number of clusters is 4 in the TCGA dataset. Error bars indicate SEM.

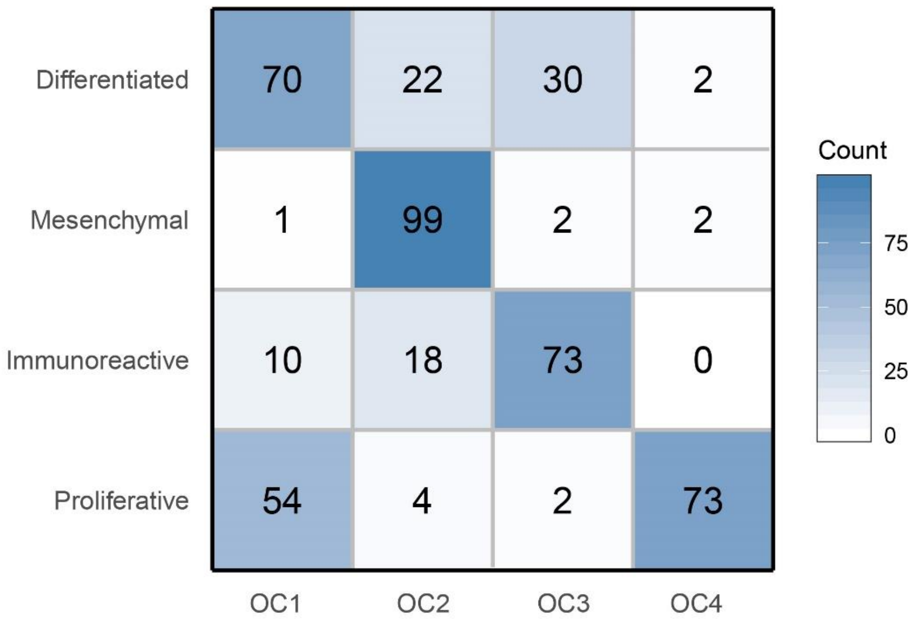

Supplementary Figure 2. A heatmap illustrating the pairwise comparison between the subtypes identified by SCCA-CC and those defined by TCGA in the TCGA ovarian cancer dataset.

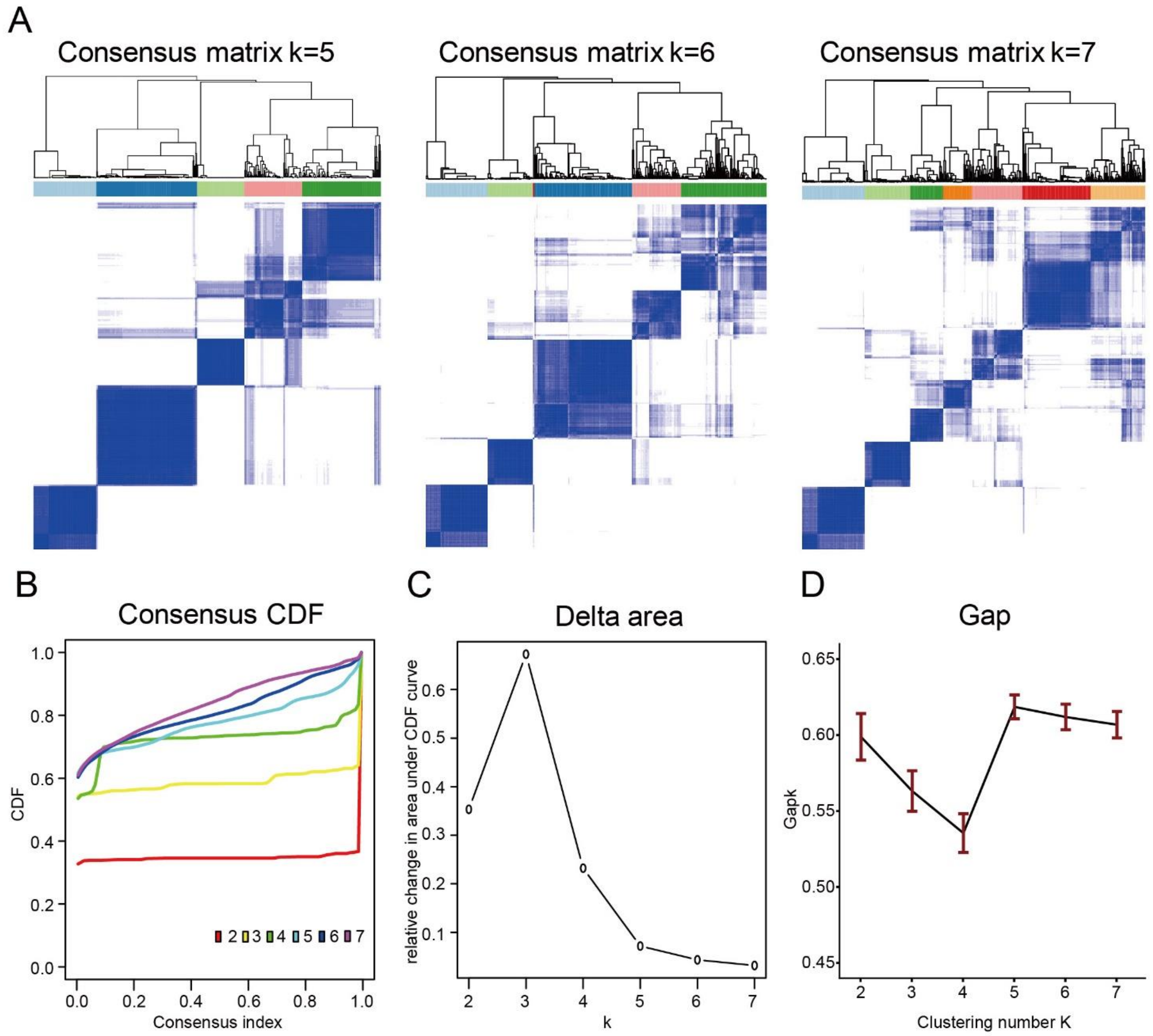

Supplementary Figure 3. Consensus clustering based on the fused data in breast cancer. **(A)** Heatmap illustrating the consensus matrices for k = 5, 6 and 7. **(B)** Consensus cumulative distribution function (CDF) plot for k varying from 2 to 7. **(C)** Delta area plot shows the relative change in the area under the consensus cumulative distribution function (CDF) curve comparing k and k – 1. At k = 5, there is no appreciable increase (Delta area < 0.1). **(D)** Gap statistic suggesting the optimal number of clusters is 5 in the TCGA dataset. Error bars indicate SEM.

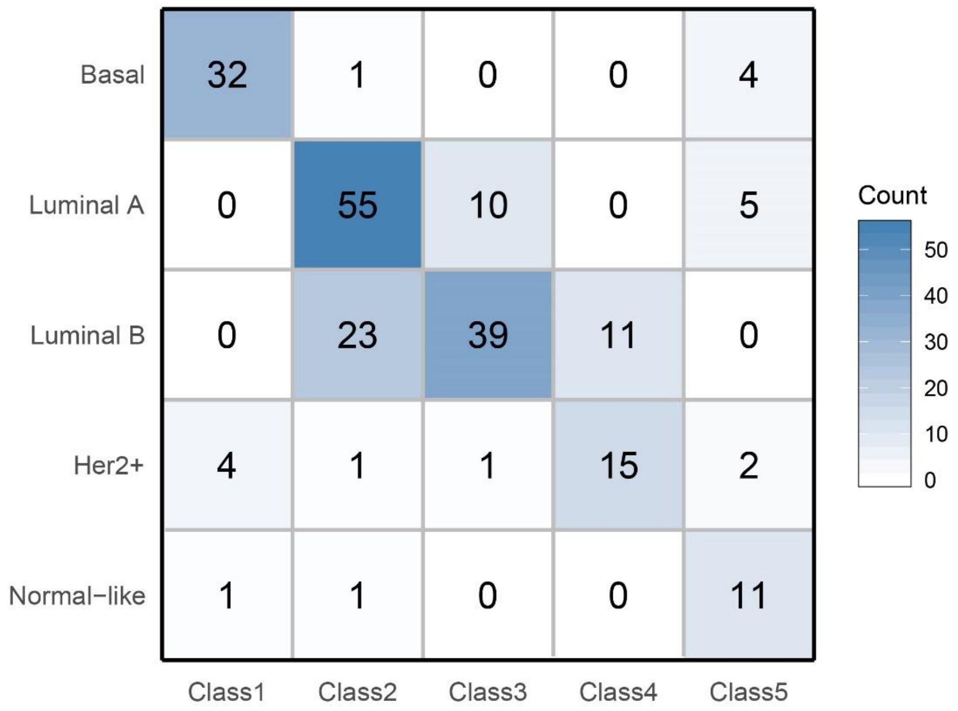

Supplementary Figure 4. A heatmap illustrating the pairwise comparison between the subtypes identified by SCCA-CC and those defined by PAM50 in the TCGA breast cancer dataset.

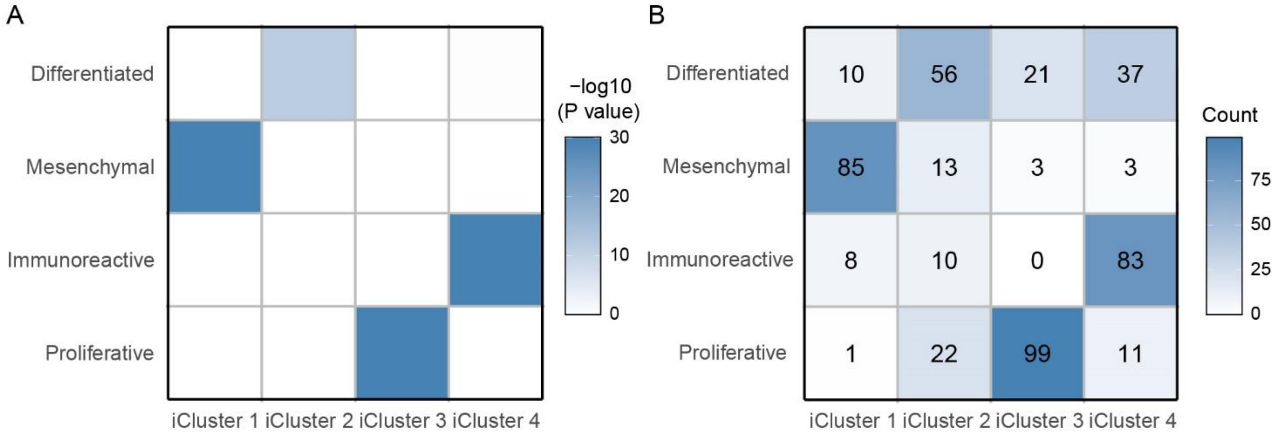

Supplementary Figure 5. Heatmaps showing the pairwise comparison between the subtypes identified by iCluster and TCGA in the TCGA ovarian cancer dataset. **(A)** The statistical significance of association quantified by hypergeometric tests. **(B)** The detailed confusion matrix.

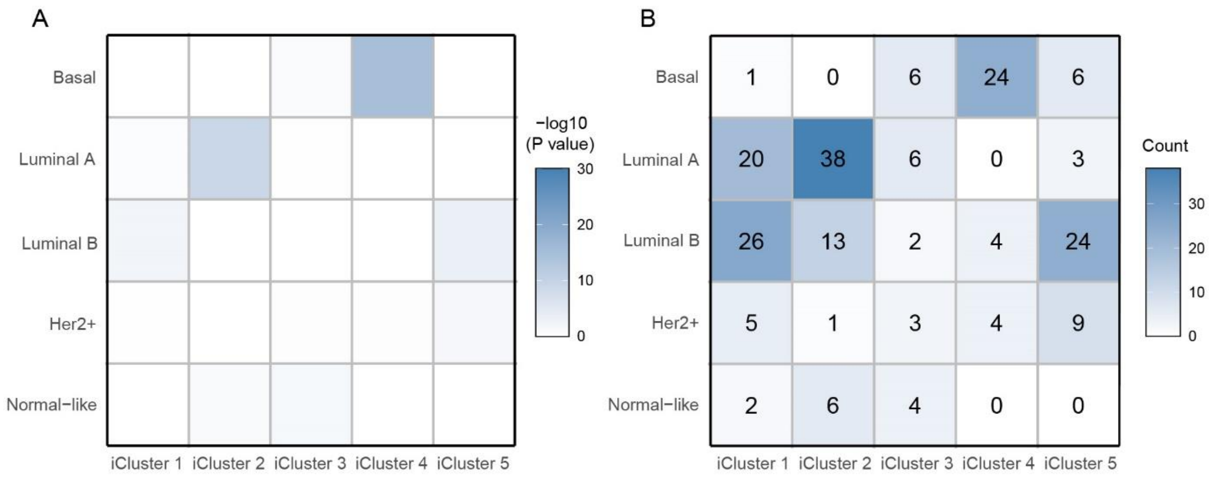

Supplementary Figure 6. Heatmaps showing the pairwise comparison between the subtypes identified by iCluster and PAM50 in the GSE22219 breast cancer dataset. **(A)** The statistical significance of association quantified by hypergeometric tests. **(B)** The detailed confusion matrix.
